# Macrophage-mediated nutrient recycling: evolutionary insights into the metabolic role of professional phagocytes

**DOI:** 10.3389/fimmu.2026.1882514

**Published:** 2026-06-24

**Authors:** Gabriela Krejčová, Adam Bajgar

**Affiliations:** 1 Department of Molecular Biology and Genetics, Faculty of Science, University of South Bohemia, České Budějovice, Czechia; 2 Institute of Entomology, The Biology Centre of the Czech Academy of Sciences, České Budějovice, Czechia

**Keywords:** coelomocytes, efferotabolism, hemocytes, innate immunity, macrophages, nutrient recycling, nutritive phagocytosis, phagocytes

## Abstract

Removal of senescent and damaged cells is fundamental for tissue homeostasis. While macrophage recognition and clearance of apoptotic cells are well characterized, the ultimate fate of the digested material remains poorly understood. Here, we explore current knowledge on the fate of engulfed material and examine how the metabolic nature of engulfed cargo shapes downstream signaling and phagocyte polarization. We also discuss emerging evidence that macrophages act as metabolic hubs, recycling and supplying nutrients to surrounding tissues. Drawing on studies in invertebrate phagocytes, we explore the evolutionary origins of this “nurturing” function and highlight its conservation in mammals, emphasizing its physiological relevance and potential contributions to metabolic disease.

## 1 Introduction: cell death as an essential component of health

Tissues of multicellular organisms undergo continuous regeneration, in which damaged, aged, or functionally exhausted cells are replaced by new ones (1). This process not only preserves the cellular capacity required for proper tissue and organ function, but also facilitates the removal of accumulated harmful and potentially toxic molecules. Cellular turnover is therefore not merely a matter of numerical homeostasis, but serves as a fundamental mechanism of tissue quality control (2).

To this end, cells are equipped with surveillance mechanisms that detect cellular stress, loss of homeostasis, or functional decline beyond a tolerable threshold. Recognition of such compromised cellular fitness or integrity leads to engagement of pathways that ultimately lead to regulated cell death (3). The precise molecular details of these processes, spanning apoptosis, autophagic cell death, ferroptosis, necroptosis, pyroptosis, and related pathways, have been extensively reviewed elsewhere, and will not be discussed in detail here (4, 5).

Typically, apoptotic cells release soluble “find me” signals, which recruit effector cells responsible for engulfing and eliminating the dying cells and cellular debris through recognition of “eat me” signals (6). This task is carried out primarily by professional phagocytes such as macrophages, which not only remove the cellular remnants, but also safely sequester and degrade them within phagolysosomes. In some contexts, non-professional phagocytes, such as neighboring epithelial cells or astrocytes, can perform these functions, despite their primary roles being unrelated to phagocytosis (7, 8).

When considered quantitatively, the scale of this process is striking. Although largely hidden from view, cellular recycling represents a massive, continuous metabolic flux. It is estimated that approximately 3x10^11^ cells are turned over daily in the average healthy human body, amounting to roughly 80 grams, with the hematopoietic system and gut epithelial cells contributing the largest fraction (9). From a purely caloric perspective, the energy contained in dying cells may appear negligible. However, from a biological standpoint, discarding these cells would be equivalent to losing valuable micronutrients and biosynthetic precursors on a daily basis.

Beyond routine tissue maintenance, cell death also accompanies episodes of profound tissue remodeling. Such remodeling occurs in physiological contexts, for example during metabolic adaptation in adipose tissue (e.g., during fasting, caloric restriction, high-calorie diet, or cold exposure), but is especially prominent during embryogenesis and development, where precisely timed, hormonally controlled cell elimination is indispensable (10–13). Classical examples include limb patterning, tail resorption, and Müllerian/Wolffian duct regression, all of which rely on developmentally programmed apoptosis coordinated by morphogen gradients, transcriptional networks, and endocrine signals (14–18).

Even more dramatic morphological transformations are observed during post-embryonic development in many animals, most notably during amphibian and insect metamorphosis (19–21). In holometabolous insects, this process reaches an extreme: the vast majority of larval tissues undergo histolysis, while most adult structures form *de novo* from imaginal discs (22, 23). This raises a fundamental question: What happens to the enormous quantity of cellular material generated during such large-scale tissue destruction? This issue becomes even more pronounced in developing embryos or metamorphosing organisms, where inefficient recycling of cellular material result in catastrophic nutrient loss accumulated during earlier life stages (24). In such contexts, cell death without subsequent nutrient recovery represents an unsustainable metabolic waste.

Whether during daily cellular turnover or large-scale developmental remodeling, macrophages occupy a central role as executors of corpse clearance (20, 25). Surprisingly, despite the apparent importance of this function, relatively little is known about how macrophages store, exploit, or recycle metabolites derived from engulfed cells (20). Given that the challenge of apoptotic cell clearance and recycling is likely shared by virtually all multicellular organisms, valuable insights may be gained from studying amoeboid phagocytic cells in evolutionarily simpler organisms (26–28).

In this article, we synthesize current knowledge on the fate of engulfed material within phagosomes and examine how the metabolic nature of the cargo directly shapes downstream signaling pathways, transcriptional reprogramming, and phagocyte polarization, providing a mechanistic basis for the concept that “you are what you eat” at the cellular level. We further review compelling evidence for nutrient recycling by phagocytic cells in invertebrate metazoans. These comparative insights help illuminate the evolutionary origins of macrophage-mediated nutrient recycling and reveal fundamental principles underlying this widespread yet understudied biological phenomenon. Building on our understanding of the recycling and biosynthetic functions of phagocytic cells, we highlight evidence that these capacities are conserved in mammalian macrophages and underscore their physiological importance by illustrating how their disruption contributes to disease.

## 2 From destruction to nutrition: the phagolysosome as a nutrient-recycling organelle

Once the phagocytic cell encounters its target, several variables influence the dynamics of phagocytosis, including the size of the internalized particle and its chemical composition, such as the presence of rigid cell walls, specialized membranes, intracellular organelles, or sterols (29–31). Moreover, the nature of the engulfed particle shapes downstream signaling cascades. For instance, phagocytosis of apoptotic cells (termed efferocytosis) typically elicits an anti-inflammatory response, whereas phagocytosis of bacteria leads to slower digestion to preserve bacterial antigens for presentation and to promote inflammatory signaling (30, 32). In line with this, while M2 macrophages possess highly acidic phagolysosomes to facilitate efficient digestion, the phagolysosomal lumen of M1 macrophages remains close to neutral (33). Despite differences in the surface receptors engaged during uptake, as well as variations in phagolysosomal maturation kinetics, luminal acidification, and the repertoire of recruited lytic enzymes, the overall progression of phagocytosis follows a fundamentally conserved sequence of events (32).

The phagocytic process is initiated by the recognition of specific ligands on the surface of the target particle by pattern recognition receptors on phagocytes. The recognized molecules are pathogen-associated molecular patterns (PAMPs), typically displayed on bacterial and fungal pathogens and damage-associated molecular patterns (DAMPs) that mark damaged host cells (34). Certain literature also distinguishes apoptotic cell-associated molecular patterns (ACAMPs), displayed on cells that undergo programmed cell death. Typically, these molecules include phosphatidylserine, translocated intracellular proteins, or modified surface structures. Recognition can be further facilitated by opsonins, such as antibodies and complement molecules in jawed vertebrates or thioester-containing proteins (TEPs) in arthropods (34–36).

Once the particle is recognized and bound, a cascade of signaling events is initiated, including the recruitment of phosphoinositide 3-kinase (PI3K) and activation of Rho family GTPases such as Rac1 and Cdc42, which drive F-actin polymerization beneath the site of attachment. This actin remodeling promotes plasma membrane reorganization and the extension of pseudopods around the particle, ultimately leading to the formation of a phagocytic cup (37, 38). Upon closure of the phagocytic cup via membrane scission, the engulfed particle becomes enclosed within a newly formed vesicular compartment known as the primary phagosome (39).

The primary phagosome is initially relatively inert. To acquire degradative capacity, this compartment must undergo a stepwise maturation process driven by regulated fusion events with components of the endocytic pathway. The dynamics of this process are controlled by Rab GTPases, which coordinate the sequential interactions of the nascent phagosome with early and late endosomal compartments, ultimately forming the late phagosome (40).

Through these interactions, the phagosome undergoes luminal acidification via acquisition of V-ATPases. The majority of degradative capacities are acquired during transformation of the late phagosome into phagolysosome via its fusion with another vesicular compartment, the lysosome (41). To facilitate this process, the late endosome migrates along microtubules toward the microtubule-organizing center (42). Lysosomal acidification is a key prerequisite for the activation of a wide range of lysosomal hydrolases, the denaturation of proteins, the release of metal ions, and the regulation of pH-dependent transporters (43). In addition, highly acidic (as low as 4.5-5) and oxidative environment represents one of the principal mechanisms that restrict further growth and survival of endocytosed bacteria within the phagolysosomal lumen (44).

This plasticity of phagolysosomal maturation is also exploited by intracellular bacterial pathogens, which actively remodel the phagosomal compartment to promote their intracellular survival. For example, *Salmonella enterica* interferes with phagosome-lysosome fusion (45), and *Mycobacterium tuberculosis* prevents phagosomal acidification by inhibiting recruitment of the vacuolar ATPase (46).

### 2.1 Enzymatic processing of phagolysosomal cargo

Since engulfed cells are highly complex structures containing many macromolecules, the lumen of the phagolysosome harbors a diverse repertoire of degradative enzymes, many of which are specialized for the phagolysosomal environment and exhibit optimal activity at acidic pH, thereby ensuring the efficient degradation of the macromolecules that constitute both prokaryotic and eukaryotic cells. Of note, many enzymes involved in phagolysosomal processing of pathogens also have roles in digestion, suggesting a connection between digestion and defense strategies (47).

Nucleic acids, present in virtually all cells, are cleaved by DNases and RNases into nucleotides. Interestingly, certain hematopoietic cells cannot efficiently synthesize nucleotides *de novo* and must therefore rely on extracellular nucleoside uptake or on recycling of phagolysosome-derived nucleosides via transporters such as the *equilibrative nucleoside transporter 3* (*Ent3*) (48). For transport from the phagolysosome, nucleotides are first dephosphorylated to nucleosides (49). Thus, nucleases in phagolysosomes are not only crucial for degradation of engulfed nucleic acids but also for their subsequent utilization by the phagocyte via the salvage pathway.

Another abundant class of macromolecules present in phagocytic targets is proteins, which are degraded by proteases into polypeptides and amino acids. Peptide bonds are cleaved primarily by cathepsins (cysteine, aspartate, and serine proteases) as well as by other endopeptidases, yielding peptides typically ranging from two to ten amino acids in length (42). These peptides are further processed into free amino acids, which can be transported into the cytosol via solute carrier (SLCs) transporters, membrane-bound proteins also implicated in nutrient sensing (50). Consequently, it has been speculated that phagolysosomal amino acid accumulation following engulfment of protein-rich apoptotic cells may contribute to intracellular nutrient sensing and the induction of satiety-like signaling pathways (32).

Lipids are also a diverse group of molecules that the phagocyte must contend with. Most of the lipid burden comes from the membranes i.e., plasma membrane of the engulfed cell and membranes of organelles. While these membranes must be degraded, the phagolysosomal membrane must remain intact to avoid damaging the cytosolic contents. However, after fulfilling its role, the limiting membrane of the phagolysosome must also be absorbed through the formation of intraluminal vesicles (51). The most prevalent lipids of membranes are phospholipids, which are cleaved by phospholipases into lysophospholipids and further to free fatty acids and phosphoglycerol. Bacterial cardiolipin is hydrolyzed by lysosomal phospholipase A2 into lysocardiolipin, which is then transported to the cytosol (52). Sphingolipids are extracted from membranes via saposins. Sphingomyelin is hydrolyzed by sphingomyelinase (53). Ultimately, sphingolipid catabolism converges on ceramide, which is hydrolyzed by ceramidase to generate sphingosine. Sphingosine can then be transported to the cytosol, where it may be phosphorylated to sphingosine-1-phosphate, a bioactive signaling molecule. Alternatively, sphingosine can be reacetylated to generate ceramide or further degraded to glycerolphosphate (53, 54).

Lysosomal acid lipase hydrolyzes cholesteryl esters derived from apoptotic cells, generating free fatty acids and unesterified cholesterol (55). In contrast to certain bacteria, animals lack enzymes capable of degrading the sterol ring, reflecting an evolutionary strategy to recycle cholesterol for essential functions such as membrane maintenance, steroid hormone synthesis, bile acid production, and lipid raft formation (56). Consequently, cholesterol is exported from the phagolysosome via the Niemann–Pick type C (NPC1/NPC2) transport system (57). When the export capacity is exceeded, cholesterol accumulates within macrophages, leading to foam cell formation and promoting a pro-inflammatory phenotype (55).

Triglycerides stored in lipid droplets of phagocytosed cells are also cleaved by lysosomal acid lipases, ultimately leading to the generation of glycerol and free fatty acids (58). Fatty acids may be then subjected to β-oxidation or re-esterification into triacylglycerol, while glycerol may eventually enter glycolysis or gluconeogenesis (59).

Glycosidic bonds within polysaccharides and glycoproteins are cleaved by glycosidases, hexosaminidases, mannosidases, and glucosidases, producing low-molecular-weight oligosaccharides and monosaccharides. Monosaccharides then bind to transport proteins in the limiting membrane and subsequently enter glycolysis, the pentose phosphate pathway, or glycogen synthesis (60).

In addition to macromolecule degradation within the phagolysosome, recovery of micronutrients also requires coordinated export and cytosolic processing pathways. For example, heme released from engulfed material is exported from the phagolysosomal lumen to the cytosol, where the cytosolic/ER-associated enzyme heme oxygenase 1 (HMOX1) catalyzes cleavage of the porphyrin ring, thereby enabling iron recovery (61, 62).

Collectively, the activity of these enzymes generates a nutritionally rich mixture of degradation products, including amino acids, fatty acids, monosaccharides, glycerol, and cholesterol, as well as iron, nucleotides, phosphate, and trace elements such as zinc and copper.

### 2.2 Phagolysosome-derived metabolites shape phagocyte function

**Figure 1:**
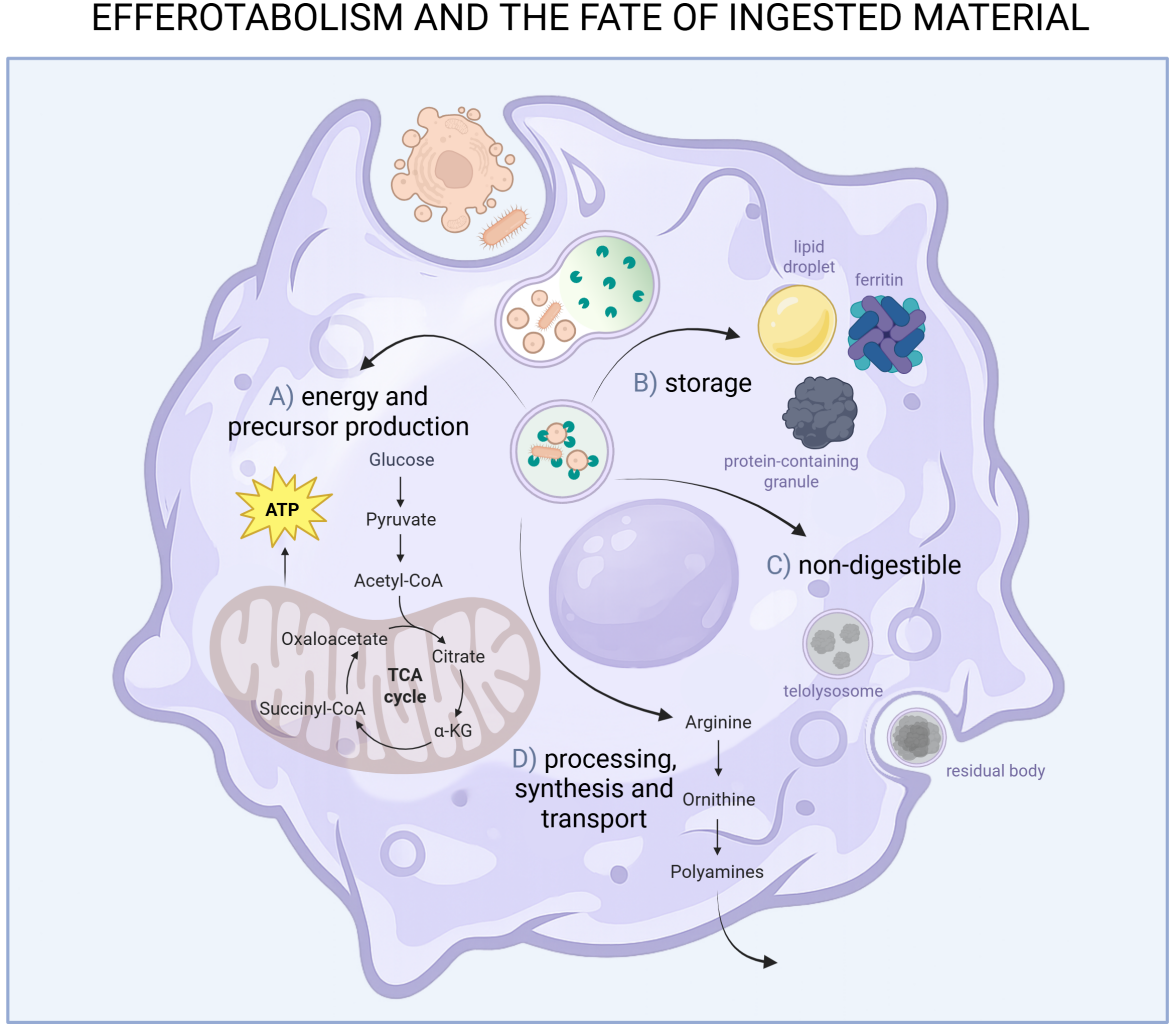
Catabolism and diverse fates of engulfed cargo in professional phagocytes. Following degradation of biomolecules within the phagolysosome, the resulting compounds can enter cellular metabolic pathways, where they are used for energy production and the synthesis of precursor molecules that support phagocyte function **(A)**. Alternatively, metabolites may be further processed and stored in specialized compartments, such as cholesteryl ester in lipid droplets or iron in ferritin **(B)**. Poorly digestible material can accumulate in telolysosomes or be expelled as residual bodies, including lipofuscin **(C)**. In addition, degradation products may be recycled into molecules destined for transport, such as arginine to polyamines **(D)**. The emerging field of efferotabolism explores how metabolic processing of engulfed cargo influences downstream signaling and transcriptional reprogramming, ultimately shaping phagocyte functional polarization. TCA, tricarboxylic acid cycle; CoA, coenzyme A; α-KG, α-ketoglutarate.

As mentioned above, degradation products can be transported from the phagolysosomal lumen into the cytosol, where they can enter downstream metabolic pathways. These include central metabolic cascades such as glycolysis, the pentose phosphate pathway, nucleotide salvage pathways, the tricarboxylic acid cycle, as well as lipid biosynthesis and steroidogenesis (63) (**Figure 1A**). Hence, the phagocyte can directly utilize the phagolysosome-derived metabolites to meet its own energetic and metabolic demands (64). Consequently, the phagolysosome is not merely a degradative organelle dedicated to the disposal of cellular debris; rather, digestion of engulfed material actively shapes downstream immunometabolic signaling and drives transcriptional reprogramming (65).

Recently, the term “efferotabolism” has been introduced to define the process by which macrophages break down, metabolize, and respond to apoptotic cell-derived macromolecules (66). Proteolytic degradation generates amino acids that are sensed at the lysosomal membrane to regulate mTORC1 activity, thereby promoting anabolic metabolism (67). Zhang and colleagues suggested that metabolic substrates derived from apoptotic cells directly influence macrophage polarization (68). Similarly, it has been documented that apoptotic cell-derived methionine is exploited for methylations to eventually fuel the production of prostaglandin E_2_ and transforming growth factor beta 1 (TGF-β1), thus demonstrating that amino acids derived from the engulfed cargo induce macrophage anti-inflammatory polarization and tissue resolution (69). Hydrolysis of cholesteryl esters releases free cholesterol, which activates liver X receptors (LXRs) and induces the expression of cholesterol efflux genes, including ATP-binding cassette (ABC) transporters (70). Liberated fatty acids can engage peroxisome proliferator–activated receptors (PPARs), supporting efferocytosis, enhancing oxidative metabolism, and promoting anti-inflammatory polarization (71). Sphingomyelin catabolism yields bioactive sphingolipid metabolites, such as sphingosine-1-phosphate, which regulate immune cell migration and modulate inflammatory signaling (72). Iron released from hemoglobin or ferritin influences hypoxia-inducible factor 1 (HIF-1α) stabilization, reactive oxygen species production, and macrophage functional programming (73). Nucleosides generated from nucleic acid degradation fuel nucleotide salvage pathways, supporting proliferation, repair, and metabolic homeostasis (74). Thus, it is evident that the biochemical composition of the engulfed cargo directly influences the functional state adopted by the phagocyte. However, this phenomenon has only begun to receive attention in recent years, and the field of efferotabolism is poised for significant growth.

Phagocytic cells can exploit not only nutrients derived from engulfed apoptotic cells, but also from bacterial pathogens. Employing stable isotope-labelled bacteria, it has been documented that phagolysosomal degradation of bacteria provides carbon atoms and amino acids that are recycled into various metabolic pathways, including glutathione and itaconate biosynthesis, thus skewing macrophage polarization toward a pro-inflammatory phenotype (75). Interestingly, the fueling of specific metabolic pathways depends on the viability of the microbe. Dead bacteria are enriched in cyclic adenosine monophosphate, which activates adenosine monophosphate protein kinase (AMPK) that inhibits mTORC1, eventually leading to decreased production of ROS, reduced interleukin-1β secretion compared to viable bacteria (75).

Overall, this framework helps explain why the nature of the engulfed cargo influences the dynamics of phagolysosome maturation, and the engagement of signaling and metabolic pathways, ultimately shaping macrophage functional polarization.

### 2.3 From storage to secretion: managing phagocytosed material

The preceding paragraphs document how the engulfed macromolecules are broken down and exploited by the phagocyte for its metabolic needs. Alternatively, phagocytosis-derived metabolites may be temporarily sequestered within the cell in dedicated storage structures, including lipid droplets, protein-containing granules, and ferritin complexes for iron storage (76) (77), (**Figure 1B**). Nonetheless, not all degradation products can be recycled or are nontoxic; non-digestible bacterial remnants or polysaccharides, for example, accumulate in residual bodies, which serve as temporary waste-storage organelles (78). These contents can eventually be expelled from the cell via exocytosis. When non-degradable material cannot be removed or recycled, it is permanently retained within telolysosomes, whose contents, including lipofuscin, metal complexes, and pigments, serve as markers of cellular senescence (79) (**Figure 1C**).

Additionally, and perhaps most intriguingly, phagocytosed material may be processed into forms that can be exported and made available to other cells within the organism (20). This possibility necessarily requires the engagement of dedicated synthetic and metabolic pathways that convert degraded macromolecules into transportable and biologically accessible forms. Such pathways must be tightly regulated, as they involve not only intracellular catabolism but also controlled metabolite trafficking across cellular membranes. It has been shown that arginine, derived from apoptotic cells, is directly exploited by the phagocyte for generation of ornithine, which is a precursor for polyamines. This conversion is essential for continual efferocytosis and tissue repair (80) (**Figure 1D**).

### 2.4 From nutritional phagocytosis to immune defense

The view of the phagolysosome as primarily a metabolic organelle is supported by examining its evolutionary origins and the factors driving its emergence. From an evolutionary standpoint, the emergence of the phagolysosome and its highly sophisticated functional properties clearly predates the evolution of multicellularity and, consequently, the emergence of the immune system (81, 82). Phagolysosome maturation and the associated enzymatic catabolism of endocytosed material are already observed in unicellular amoebozoans, suggesting that these processes originated in the common ancestor of Amoebozoa and Metazoa (83). As a result, striking functional parallels can be observed between the phagolysosomes of mammalian macrophages and those of free-living amoebae, such as *Acanthamoeba* (84, 85). Together with another representative of the Amoebozoa, *Dictyostelium discoideum*, these organisms have long served as experimental models for studying phagocytosis and phagolysosomal maturation (86–89). Recent comparative genomic analyses support the idea that macrophage-like phagocytic cells represent one of the most ancient metazoan cell types, highlighting the deep evolutionary roots of phagocytosis and intracellular digestion in eukaryotes (90).

The degree of evolutionary conservation of phagolysosomal function across these systems is such that several well-known human intracellular bacterial pathogens, including *Legionella pneumophila*, *Mycobacterium tuberculosis*, and *Coxiella burnetii*, employ highly similar survival strategies when confronted by free-living amoebae in the environment (91). This observation has led to the widely accepted hypothesis that, within the human host, these pathogens exploit mechanisms originally refined through evolutionary interactions with environmental amoebae, which have effectively served as a long-term “training field” for the development of phagolysosome evasion mechanisms (91). Given these striking similarities between phagocytosis in amoebae and mammalian phagocytes, these single-cell eukaryotes have become established models for investigating how intracellular pathogens, including *Mycobacterium* and *Legionella*, evade phagolysosomal killing and persist within phagocytic host cells (92, 93).

It is therefore unlikely that the original function of the phagolysosome was the elimination of bacteria as potential threats to a multicellular organism. Rather, its primordial role was most likely the phagocytosis and intracellular digestion of bacteria and unicellular eukaryotes for nutritional purposes (81). This process, commonly referred to as nutritional phagocytosis, can still be readily observed in the digestive systems of many multicellular organisms, with the notable exception of vertebrates or arthropods (94). In these groups, extracellular digestion became the dominant strategy for nutrient acquisition, thereby reducing, but not eliminating, the nutritional relevance of intracellular digestion. It was Élie Metchnikoff who postulated that macrophages are evolutionarily derived from enteric phagocytes (95). In most invertebrates, enteric phagocytes, representing one of the two major cell types of the intestine, also behave “professionally” in terms of phagocytosis, and are responsible for intracellular digestion of food particles (94). Hence, it has been proposed that the digestive and immune systems have a common origin (47).

It can thus be inferred that the ancestral macrophage-like cells of early multicellular organisms were already well equipped to digest both bacterial and eukaryotic cells within the phagolysosome (82). This pre-existing digestive capacity was subsequently co-opted during evolution for immune defense, enabling the elimination of bacteria that breached primary protective barriers, as well as for the clearance of damaged or dying cells within multicellular tissues (81).

In unicellular organisms, the benefits of nutrient acquisition through phagocytosis are largely cell-autonomous (83). In contrast, multicellularity introduces new constraints and opportunities, including the need to redistribute resources between different cell types, tissues, or developmental compartments (96). Under these conditions, phagocytes are no longer merely consumers of engulfed material, but instead play an important role in converting cellular debris into reusable metabolic currency for the benefit of the organism as a whole (94). Experimental evidence for this type of metabolic and nutritive function of phagocytic cells has been documented across the major branches of metazoan evolution.

## 3 Nutritive role of professional phagocytes in early-diverging metazoans

All living organisms depend on the external intake of nutrients to meet their immediate energetic demands and provide essential building blocks and micronutrients (97). Over the course of animal evolution, dedicated organs arose to coordinate nutrient acquisition, systemic allocation, metabolic processing, storage, and the mobilization of these resources during physiological stress (97). However, it seems evident that, prior to the emergence of such specialized metabolic organs, these functions may have been fulfilled by amoeboid professional phagocytes, which are functionally analogous to mammalian macrophages (94).

In the following section, we focus on documented examples of such unexpected functions in invertebrate phyla, where phagocytic cells not only mediate innate immune responses but also couple intracellular digestion with nutrient redistribution. Importantly, this metabolic role of innate immune cells is not universally assumed and is often considered secondary to their defensive function, despite growing evidence that immune and metabolic processes are tightly intertwined in early-diverging metazoans. These animal phyla were selected to represent major evolutionary branching points in animal evolution, spanning the origin of multicellularity (Porifera), the emergence of true tissues (Cnidaria), the origin of bilaterian body plans (Platyhelminthes), increasing organ-system complexity within protostomes (including Annelida, Mollusca, and Arthropoda), and the major split between protostomes and deuterostomes (including Echinodermata), which together encompass most of extant animal diversity. These systems provide valuable insight into how metabolite handling downstream of phagolysosomal degradation may have been adapted to support multicellular organization and cooperative metabolism.

### 3.1 Porifera

Nutritive and metabolic functions are already evident in the amoeboid cells of Porifera (98). These basal metazoans, positioned near the root of the metazoan phylogenetic tree, lack true tissues in the classical sense (99). With few exceptions, their bodies consist of only a limited number of fundamental cell types. Flagellated collar cells (choanocytes) are specialized for water filtration and the phagocytic uptake of suspended particles and their partial degradation. The released nutrients are utilized directly by the choanocyte to support its own metabolic demands, or alternatively they can be transferred to other sponge cell types that lack phagocytic capacity and are therefore dependent on intercellular nutrient provisioning (100). In this context, amoeboid mesohyl cells, commonly referred to as archaeocytes, act as intermediaries in nutrient distribution. Material derived from choanocyte digestion can be passed to archaeocytes either via release of partially processed phagosomes/phagolysosomes or through direct transfer, after which it is internalized by secondary phagocytosis (94, 101). These motile cells then further digest the ingested material within phagolysosomes, and the resulting metabolites are be stored in the cytosol, particularly in the form of lipid droplets and glycogen granules (102). These reserves are mobilized during periods of reduced food availability, gametogenesis, environmental stress, or wound healing. Alternatively, the ingested nutrients are used to satisfy the archaeocyte’s own energetic requirements or redistributed throughout the sponge body to support other cell types. The metabolic role of these cells is further highlighted during asexual reproduction. In freshwater sponges, reproductive structures known as gemmules contain large numbers of archaeocytes that differentiate into nutrient-rich storage cells, or thesocytes. These cells accumulate substantial reserves of glycogen, lipids, and RNA enabling long-term survival during dormancy and providing the primary nutritional support for the developing sponge upon gemmule hatching (103). Thus, archaeocytes not only sustain everyday metabolic functions but also ensure energetic continuity across life cycle transitions and adopt the function of storage organs in primitive organisms lacking specialized tissues for this purpose. The existence of certain motile cells, referred to as nurse cells, which transport lipidic yolk to developing oocytes, has been documented (104–106). These cells share many characteristics with archaeocytes - motility, amoeboid shape, phagocytic ability - making it likely that archaeocytes could serve this role (107). However, definitive identification has not yet been achieved. Upon injury, the archaeocytes actively migrate toward the wound site and clear dead cells, damaged extracellular matrix, and microbes, and subsequently, they differentiate into the required cell type to seal the wound (99).

**Figure 2:**
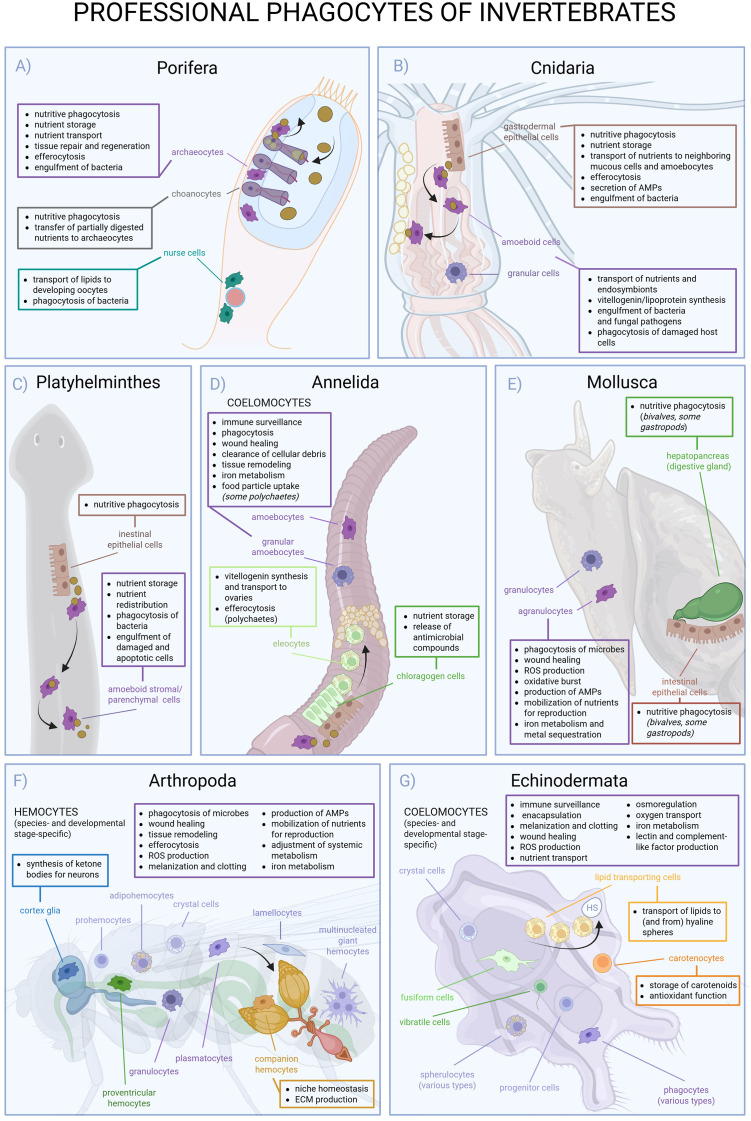
Professional phagocytes and their roles across invertebrates. In Porifera, choanocytes mediate the uptake and partial digestion of food particles, which are subsequently further digested and distributed throughout the body by archaeocytes **(A)**. In Cnidaria **(B)** and Platyhelminthes **(C)**, nutritive phagocytosis is carried out by gastrodermal epithelial cells, whereas amoeboid circulating immune cells contribute to nutrient transport in addition to their immune functions. Besides classical phagocytic coelomocytes, annelids possess chloragogen cells, from which eleocytes detach and participate in nutrient transport **(D)**. In molluscs, some species perform intracellular digestion, while others rely exclusively on extracellular digestion. Apart from immune-related functions, immune cells also contribute to nutrient mobilization for reproduction and iron metabolism **(E)**. In Arthropoda, a diverse range of hemocytes has been described, some exhibiting features suggestive of tissue-residency **(F)**. A similarly diverse set of coelomocytes is found in Echinodermata, with some fulfilling nutritive roles **(G)**. AMPs, antimicrobial peptides; ECM, extracellular matrix; ROS, reactive oxygen species.

Overall, archaeocytes function as central hubs of nutrient processing, storage, and allocation in the absence of specialized circulatory or transport systems. The high functional plasticity of archaeocytes allows them to simultaneously support tissue repair, cellular differentiation, regeneration, and overall organismal homeostasis (108, 109), (**Figure 2A**).

### 3.2 Cnidaria

A comparable functional integration is observed in Cnidaria, in which nutritive phagocytosis is performed by gastrodermal epithelial cells (110). This phenomenon is particularly well documented in *Hydra*, where the gastrodermal epithelial cells accumulate energy reserves such as lipids and glycogen in their cytosol after food uptake (111, 112). Interestingly, these nutrients can be exploited by the neighboring mucous cells or transferred to amoebocytes, which transport them through the mesoglea, a pattern reminiscent of the intercellular nutrient redistribution observed in Porifera (111, 113, 114). These same epithelial cells are also responsible for efferocytosis, which becomes especially pronounced during head regeneration (115). Yet, the impact of efferocytosis in head-regenerating tips on the regenerative process might not be restricted solely to a scavenging function, but may also trigger the developmental function of the endodermal cells, which at that time start developing an organizer activity (112). Gastrodermal epithelial cells also possess immune defense strategies, with the signaling cascades being dissected more elaborously than in Porifera (116, 117).

In cnidarians, motile amoeboid cells are represented by amoebocytes. Several studies pointed to the role of these cells in nutrient transport via the mesoglea, as they contain extensive glycogen depots and smaller inclusions of lipid droplets (114, 118–120). They also incorporate carbon and nitrogen derived from fixation by photosynthetic endosymbionts of jellyfish, thus functioning as intermediate metabolic carriers. Interestingly, they also physically carry and spatially transport endosymbionts to light-rich surfaces (120). It has also been documented that amoebocytes uptake lipids from the gastrodermis and process them into transportable lipoprotein-like particles (vitellogenin/apolipoprotein B-like complexes), which are subsequently internalized by oocytes (118). Their antimicrobial function also relies on actin remodeling of the cytoskeleton and acidification of the phagolysosome (121). Although is unknown whether these cells are derivatives of the gastrodermis or arise from a different cell lineage, it is evident that both gastrodermal epithelial cells and amoebocytes represent a cell type with both nutritive and immune functions (**Figure 2B**).

### 3.3 Platyhelminthes

In Platyhelminthes, intestinal epithelial cells (gastrodermal cells) digest food obtained from the extracellular environment through both extracellular and intracellular processes (122, 123). Cells displaying signatures of macrophages are located in the stroma (124). These motile stromal/parenchymal cell phagocytose experimentally applied beads, and contain phagolysosomes and nutrient storage organelles (94, 125). They were shown to take up algae engulfed by enteric phagocytes, suggesting that they also take up nutrients that have been digested and partially absorbed by gut epithelial cells, and presumably help to conduct them to distant tissues by bulk cytoplasmic continuity and diffusion (124, 126). This notion is further supported by frequent occurrence of vesicles, which are presumably derived from gastrodermal cells (125). However, direct experimental evidence demonstrating nutrient transfer, such as tracer studies using labeled bacteria or food particles to follow the distribution and sharing of metabolites between digestive and stromal cells, has not yet been obtained. Current interpretations are therefore largely based on extensive morphological and ultrastructural observations showing close physical associations between digestive epithelial cells and motile parenchymal phagocytes (127). These motile amoeboid cells are frequently found in intimate contact with surrounding cell types whose metabolic supply would otherwise be difficult to explain solely by passive diffusion, a mechanism that is unlikely to provide tightly regulated or efficient nutrient distribution (e.g., neurons, muscles, glands) (128–130). They may phagocytose small debris or apoptotic cells, but not bulk food from the gut lumen (131). Freshwater planarians are well-established experimental models for regeneration, as they are capable of restoring an entire organism from small tissue fragments (132) (133). Notably, following injury, stromal/parenchymal phagocytic cells accumulate at wound sites, where they engulf damaged and apoptotic cells and contribute to tissue clearance (131) (**Figure 2C**).

### 3.4 Annelida

In annelids, digestion takes place predominantly extracellularly within the lumen of a fully developed digestive tract (94, 110). The intestinal enterocytes do not take up nutrients via phagocytosis in most annelid species; however, in some polychaetes (e.g., *Arenicola marina*) they retain the ability to uptake food particles that are then transferred to wandering amoebocytes, which participate in particle uptake and processing (134, 135).

A particularly noteworthy organ is the chloragogen tissue, which functions as an analogue of both the liver and adipose tissue (136). This tissue forms a dense layer surrounding the intestine, where it accumulates substantial reserves of lipids, glycogen, and proteinaceous granules (137). Interestingly, chloragogen cells can detach from the gut epithelium and enter the coelomic circulation as eleocytes (138). This detachment appears to be a normal part of physiological turnover and is enhanced during reproductive maturation (139). Notably, eleocytes have been shown to synthesize vitellogenin and contribute to ovarian maturation, highlighting their role in reproductive nutrient provisioning (140–142). Although injury and immune challenge alter the populations of circulating cells, direct mechanistic evidence for specific stress-induced detachment signals remains limited. Chloragogen cells do not appear to engage in phagocytosis, but are considered a defense tissue since they contain enzymes and lysosomes, release compounds with antimicrobial activity, and participate indirectly in immune responses by producing humoral factors (143). While in eartworms the eleocytes do not seem to be phagocytic, in polychaetes they engulf the disintegrating muscle cells during metamorphosis (144).

Cells freely circulating in the coelomic cavity are collectively referred to as coelomocytes, and apart from chloragogen cells/eleocytes, they also comprise amoebocytes and granular amoebocytes (granulocytes) (145). Amoebocytes are highly motile and are primarily associated with immune surveillance, phagocytosis, and wound responses, whereas chloragocytes and/or eleocytes play predominantly metabolic and storage-related roles (145, 146). Amoebocytes are also thought to participate in the clearance of cellular debris and tissue remodeling processes, as they migrate toward apoptotic and damaged tissues (147, 148). In addition, they likely contribute to the regeneration of body parts, as experimental coelomocyte depletion results in significantly impaired cell division and blastema formation during regeneration (147). However, direct recycling of nutrients to regenerating somatic tissues has not been experimentally demonstrated. Additionally, amoebocytes appear to be directly involved in iron metabolism, as they have been shown to store ferritin in *Eisenia andrei*. Notably, following infection with *E. coli* and *B. subtilis*, ferritin expression is strongly upregulated, thus suggesting a functional role in the innate immune responses against both Gram-positive and Gram-negative bacteria (149) (**Figure 2D**).

### 3.5 Mollusca

In Mollusca, professional phagocytes are considered to be hemocytes, amoeboid macrophage-like cells circulating in the hemolymph (150–152). Those containing granules are denoted as granulocytes, while those lacking granules are referred to as agranulocytes or hyalinocytes. In some species, temporary or intermediate forms have been described, suggesting a significant degree of plasticity. These cells actively phagocytose microorganisms, degrade internalized material through lysosomal pathways, migrate to wounds, produce ROS, undergo an oxidative burst, and release antimicrobial peptides (151, 153–158). Interestingly, some antimicrobial peptides are produced only by specific hemocyte subtypes, suggesting functional diversification (159). Molluscan hemocytes contain substantial amounts of glycogen and lipids, whose composition and quantity change significantly in response to stressors such as nanoparticle exposure (160). Their role in energy metabolism and as significant nutrient reservoirs is further supported by a study indicating that nutrient reserves in hemocytes are mobilized for reproduction, leaving less available for hemocyte energy metabolism and defense functions. Although no study has explicitly demonstrated that hemocytes deliver nutrients to developing oocytes (such as vitellogenin transfer in Annelida), the results are consistent with a trade−off between reproductive energy demand and immune/metabolic capacity in hemocytes, supporting the idea that lipid metabolism in hemocytes is connected to life−history stage (161, 162).

Hemocytes are also involved in the metabolism of heme. Similarly to annelids, immune cells of molluscs express ferritin, and this expression is upregulated in response to bacterial challenge, suggesting that hemocytes participate in iron metabolism during infection (163). This is reminiscent of nutritional immunity in vertebrates, in which the host limits pathogens’ access to iron. Hemocytes are also involved in the handling of other metals, such as copper, zinc, and cadmium (164, 165) (**Figure 2E**).

### 3.6 Arthropoda

Arthropod hemocytes include several types, such as plasmatocytes, granulocytes, crystal cells, lamellocytes, oenocytoids, multinucleated giant hemocytes, spherule cells, or adipohemocytes. However, not all of these cell types are phagocytic: some specialize in processes such as melanization or encapsulation. In most species, the primary phagocytic hemocytes are plasmatocytes and/or granulocytes (166). Importantly, for some hemocyte types the boundaries between immune and metabolic functions are blurred. As their name suggests, adipohemocytes are circulating cells characterized by abundant lipid droplets, which occupy the majority of their cytoplasm, and sometimes glycogen stores, suggesting their primary function in energy storage (167, 168). Similar large electro-lucent spherules were also described in some spherulocytes (169). Single-cell RNA profiling of *Drosophila* hemocytes has also identified a subpopulation of adipohemocyte-like cells characterized by high expression genes associated with lipid metabolism and starvation responses (170). In addition to lipid storage, hemocyte lipid-handling capacity may also contribute to systemic lipid clearance and redistribution, as recently shown *in vivo* (171).

The most well-characterized example of metabolic recycling and nutrient provisioning by phagocytes has been described during metamorphosis in *Drosophila melanogaster*. Genetic ablation of macrophage-like plasmatocytes results in pupal lethality even under axenic conditions, indicating that their essential role during metamorphosis is linked to tissue remodeling rather than the clearance of microbes potentially leaking from the gut (20, 172). During this stage, plasmatocytes actively engulf degenerating larval tissues through macroendocytosis and efferocytosis, with a particular contribution to the clearance of adipocyte and muscle debris (25) (173). It has been shown that they produce lipoproteins that are required for the proper development of adult structures, including the ovaries, and for early fecundity, thereby fulfilling a critical nutritive function (20). Furthermore, although vitellogenin production has traditionally been attributed exclusively to the fat body, accumulating evidence indicates that macrophage-like cells can also express this key precursor of yolk proteins essential for embryonic development (174, 175). Trophic roles of hemocytes have also been suggested following a blood meal in the blacklegged tick, *Ixodes scapularis*, which induces broad metabolic reprogramming of hemocytes, alongside an overall increase in hemocyte numbers. Single-cell RNA sequencing identified two distinct hemocytes clusters with pronounced metabolic signatures. Ablation of these clusters resulted in reduced weight of engorged ticks and impaired molting of nymphs to adults, suggesting that hemocytes contribute to successful feeding and developmental progression. Interestingly, the transcriptional profiles of these metabolically specialized clusters are further modulated upon infection (176). Consistent with this, infection-induced changes in systemic nutrient allocation mediated by hemocytes have also been described in *Drosophila*. In both adults and larvae, hemocytes release signaling molecules that drive systemic metabolic switch, which is essential for overcoming the challenge (177–179).

An elegant study from the laboratory of Tina Mukherjee further highlights the central role of hemocytes as metabolic sensors and regulators of organism-wide nutrient allocation. Upon high-sugar feeding of *Drosophila* larvae, hemocytes adopt a pro-inflammatory phenotype accompanied by a metabolic shift toward aerobic glycolysis, reminiscent of M1-like polarization observed in macrophages responding to pathogenic stimuli (177, 180, 181). This reprogramming at the cellular level has systemic consequences, as adult flies emerge with reduced body size, underscoring the role of macrophage-like cells in coordinating developmental growth with nutritional status (180).

Analogous to phagocytes in lower invertebrates, arthropod hemocytes have also been implicated in iron transport and homeostasis (182, 183).

Although not derived from the hematopoietic lineage, glial phagocytes have also been shown to perform clear trophic functions in the brain of *Drosophila melanogaster*. In particular, cortex glia have been demonstrated to mobilize their lipid stores to synthesize ketone bodies, which are subsequently supplied to neurons to support memory formation and maintenance under conditions of starvation (184). While not bona fide hemocytes, their stable tissue association, capacity for local metabolic support, and phagocytic activity highlight that key functional features attributed to tissue-resident macrophages can also arise in non-hematopoietic cell types (**Figure 2F**).

### 3.7 Echinodermata

In echinoderms, macrophage-like cells are represented by coelomocytes, mesoderm-derived cells that circulate within the coelomic fluid and exhibit pronounced amoeboid behavior. These cells are highly phagocytic and play central roles in immune surveillance, enacapsulation, clotting, melanization, and wound healing (185). The morphological and functional diversity of coelomocytes in surprisingly high. Queiroz and Custódio documented 15 coelomocyte types in sea urchins, including vibratile cells, fusiform cells, and spherulocytes (186). Morphological studies classify spherulocytes as cells containing cytoplasmic vesicles and pigments, which often include lipid-pigment complexes in sea cucumber coelomocytes, implying these cells contain lipid inclusions not exclusively associated with phagocytosis (187). Crystal cells, observed in holothuroids and occasionally in echinoids, were originally thought to be coelomocytes containing intracellular vacuoles with crystalline structures. However, a recent study showed that these cells are actually phagocytes that have engulfed uric acid microcrystals generated as metabolic waste rather than immune granules (188). Recently, a novel coelomocyte type has been identified in sea cucumbers and designated as carotenocyte. These cells accumulate carotenoids such as astaxanthin and canthaxanthin, which are dietary nutrients, thus linking nutrition and immune defense. Carotenocytes express carotenoid metabolism genes, form aggregates during immune responses, and act as antioxidant shields around immune cell clusters (189).

Histological studies of holothurian intestines have shown that coelomocytes occur near the digestive epithelium, extend basal projections through tissue layers, suggesting a role in the uptake and processing of material from intestinal cells (190). However, this study did not demonstrate that coelomocytes transport nutrients to other tissues. When pathogenic bacteria enter the gut lumen, coelomocytes are attracted toward gastrodermal cells via cytokines released from these cells (191). Additionally, coelomocytes appear to be intercalated between epithelial gut cells, and their involvement in nutritive phagocytosis has been suggested in some studies, though it has not been experimentally confirmed (192, 193). Nonetheless, coelomocytes are implicated in osmoregulation as well as oxygen and nutrient transport (186, 194, 195). Remarkably, in sea cucumber larvae, diet-derived lipids are transported from the stomach and intestine by mesenchyme cells, termed lipid transporting cells (LTC), presumably in the form of free fatty acids or lipoproteins, to the hyaline spheres. Hyaline spheres are clear, storage cells, and the lipids transported here by LTCs ale converted to triacylglycerol with a higher saturated fatty acid content. Lipid stores in the hyaline spheres are subsequently utilized during metamorphosis, while LTCs are again essential for this process, transporting neutral lipids within the blastocoel (196).

In contrast to cnidarians and annelids, where direct transport of lipids and vitellogenin to oocytes by circulating immune cells has been demonstrated, evidence for this process in echinoderms is only indirect. Nutritive phagocytes in the gonads of both sexes contain major yolk protein, which serves as a precursor for the synthesis of proteins and other molecules necessary for eggs and sperm (197). In echinoids, major yolk protein as well as it precursor, vitellogenin, are also found in the coelomic fluid and in phagocytes outside the gonad, indicating a systemic component of nutrient transport linked to coelomocyte activity (198).

Presumably one of the clearest examples of nutritional immunity in this phylum is iron handling by echinoderm coelomocytes (199). Ferritin expression is upregulated in stimulated coelomocyte, functioning as an acute-phase protein and documenting that iron sequestration during immune responses is an ancient mechanism predating vertebrates (199). One of the most striking iron-handling mechanisms involves red spherule cells, a coelomocyte subtype containing vesicles filled with the pigment echinochrome A (200). Exposure to microbial ligands or pathogens triggers degranulation of red spherule cells and the release of echinochrome A into the coelomic fluid (200). Lysates of these cells reduce bacterial colony formation, showing antimicrobial activity (200). Echinochrome acts as a metal chelator, as addition of ferric iron (Fe³^+^) suppresses its antimicrobial activity, thus depriving microbes of iron (200) (**Figure 2G**).

From the examples discussed above, it can be inferred that the ability of amoeboid professional phagocytes to degrade macromolecules within the phagolysosome became, early after the emergence of multicellularity, closely linked to their capacity to handle and redistribute nutrients within the organism (201, 202). In several early-diverging metazoans, motile cells of the body cavity often participate in nutrient storage, metabolic regulation and the distribution of metabolites, which can blur the distinction between immune and metabolic cell types (94). Such cells frequently both sense and transmit metabolic signals, contributing to systemic regulation of nutrient fluxes and, in some cases, directly supplying essential nutrients to other tissues (142). With the evolutionary emergence of specialized metabolic organs and storage tissues, these functions became increasingly compartmentalized, reducing the reliance on amoeboid phagocytic cells as the primary sites of metabolic storage (27). This transition likely facilitated the progressive specialization of phagocytes toward immune defense, although many ancestral metabolic features have been retained. The imprint of these earlier nutritive functions therefore remains evident in modern phagocytes, including mammalian macrophages, particularly within tissue-resident populations where metabolic and immune roles remain tightly integrated (203).

## 4 Nutrient-recycling functions of mammalian macrophages and their pathological implications

As discussed in previous paragraphs, phagocytosis likely evolved as a strategy for food acquisition and was later repurposed during evolution for the engulfment and elimination of pathogenic microbes (94). In mammals, phagocytosis appears to function primarily in the elimination of bacteria and is therefore commonly viewed purely as an immune defense mechanism. However, traces of the evolutionary heritage of macrophages as metabolically active, nurturing cells may still persist even in mammals.

As discussed in Section 2.2, isotope-labeled bacteria can serve as a nutrient source for macrophages, with bacterial-derived carbon and amino acids being incorporated into host metabolic pathways and contributing to macrophage bioenergetic metabolism (75). In addition, it has been experimentally documented that nutrient-deprived macrophages display enhanced phagocytosis of heat-inactivated *E. coli*, raising the possibility that macrophage may use ingested bacteria as a compensatory mechanism to meet their energy demands (204). Our understanding of the role of macrophages in the mammalian body has changed fundamentally with the recognition of tissue-resident macrophages. Most organs and tissues harbor distinct populations of functionally specialized macrophages that contribute substantially to tissue homeostasis and physiological function (203, 205). Many tissue-resident macrophage populations are established during embryogenesis, arising from progenitors in the yolk sac and later in the fetal liver, which seed developing organs in successive waves (205–207). Once established, tissue-resident macrophages perform a wide range of functions that have been extensively reviewed in the recent literature (203, 205). In the context of the perspective presented here, however, their roles in metabolic provisioning are of particular interest.

### 4.1 Reverse cholesterol transport

**Figure 3:**
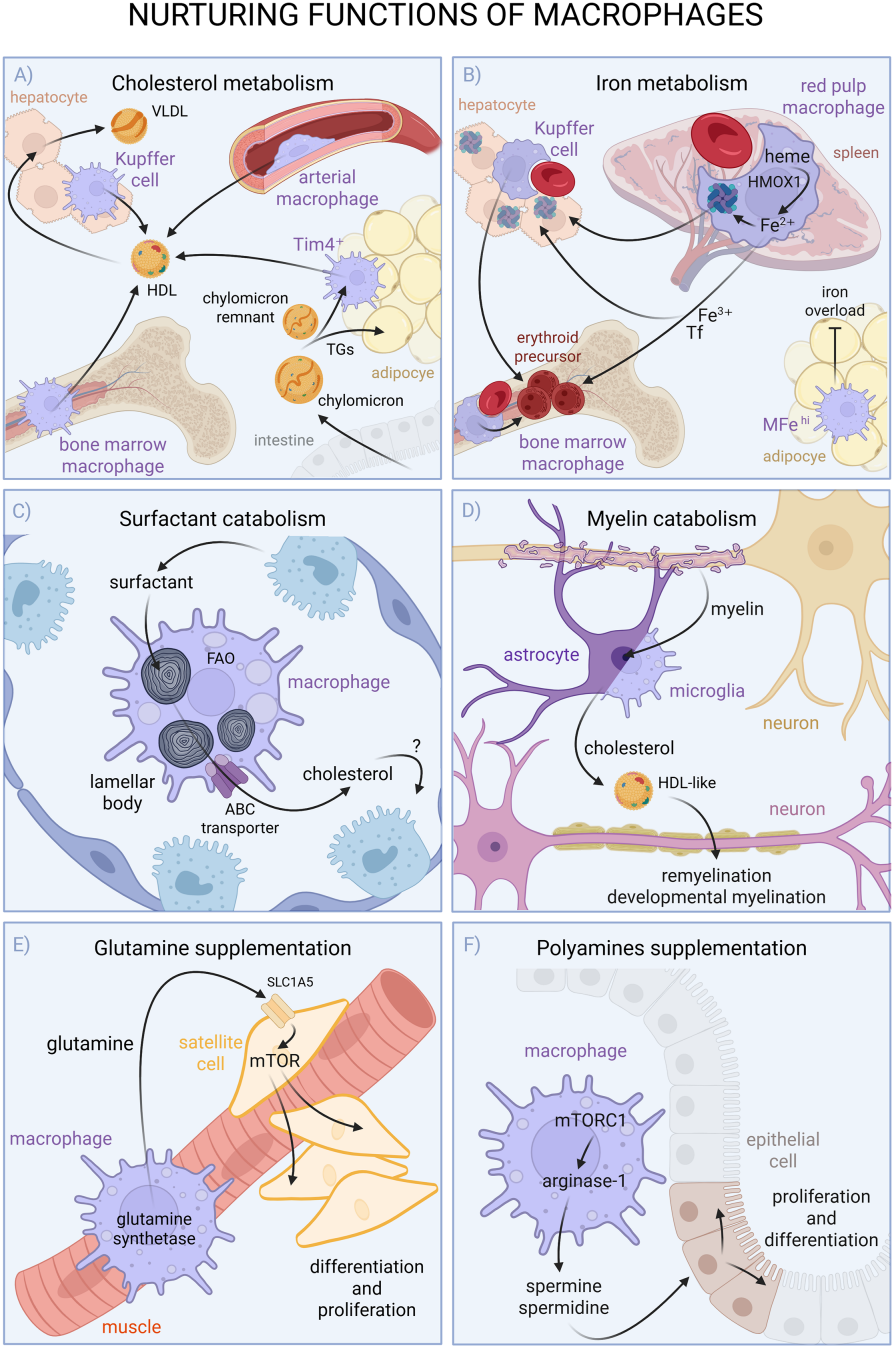
Nurturing functions of mammalian macrophages. Macrophages contribute to cholesterol recycling **(A)**, recycling of iron **(B)**, catabolism of pulmonary surfactant **(C)**, myelin degradation and subsequent remyelination of neurons **(D)**, glutamine supplementation of muscle satellite cells **(E)**, and polyamine provisioning to gut epithelial cells **(F)**. VLDL, very low-density lipoprotein; HDL, high-density lipoprotein; TGs, triglycerides; FAO, fatty acid oxidation; ABC, ATP-binding cassette; HMOX1, heme oxygenase 1; mTOR, mechanistic target of rapamycin; mTORC1, mechanistic target of rapamycin complex 1; SLC1A5, solute carrier family 1 member 5; Fe, ferrum; Tf, transferrin.

Presumably, the most well-characterized metabolic role of macrophages is their function in reverse cholesterol transport, a process by which excess cholesterol is transported from peripheral tissues to the liver, where it can be repacked into new very low density lipoproteins (VLDL) or excreted into bile and ultimately eliminated in the feces, thus acting as a crucial anti-atherogenic mechanism (208). Excess cholesterol is exported from macrophages via ATP-binding cassette transporters (ABCA1 and ABCG1) (209). Subsequently, free cholesterol is transferred to lipid-poor apolipoprotein A-I (apoA-I), the principal lipoprotein of nascent HDL. Nascent HDL particles acquire additional cholesterol, which is esterified by lecithin–cholesterol acyltransferase (LCAT) to form mature HDL. Mature HDL then transports cholesterol through the circulation back to the liver (210). Under physiological conditions, arterial macrophages contribute locally but make a relatively minor contribution compared to liver macrophages or interstitial macrophages in peripheral tissues. However, in pathological states, when macrophage cholesterol-handling capacity is exceeded, macrophages in the arterial intima transform into foam cells, a key event in the development and progression of atherosclerotic plaques (208). Recently, the contribution of adipose tissue macrophages (ATMs) to reverse cholesterol transport has also been demonstrated. Dietary lipids are absorbed in the intestine and packaged into chylomicrons, which deliver triglycerides to adipose tissue via lipoprotein lipase-mediated hydrolysis. The resulting cholesterol-rich chylomicron remnants are taken up and processed by a Tim4^+^ resident subpopulation of ATMs, which upregulate lysosomal lipid processing postprandially and mediate cholesterol efflux to HDL (211). In this context, ATMs act as local lipid-processing hubs, facilitating cholesterol recycling after nutrient intake (**Figure 3A**). Nonetheless, whether cholesterol derived from engulfed apoptotic cells is efficiently mobilized by macrophages and routed into reverse cholesterol transport pathways remains to be determined.

### 4.2 Iron recycling

While macrophage-mediated cholesterol efflux is central to lipid homeostasis, these cells also play an equally critical role in iron recycling. Approximately 2–3 million erythrocytes must be cleared from the human circulation every second, as their average life span is only about 120 days (212). This corresponds to roughly 200 billion red blood cells per day, representing a substantial turnover. Notably, macrophages are not only responsible for their removal but also for iron recycling, as dietary iron availability is limited (213). The principal cells involved in erythrophagocytosis and iron recycling are splenic red pulp macrophages. Following erythrophagocytosis and phagolysosome formation, erythrocytes are degraded by ROS and lysosomal enzymes, leading to the release of hemoglobin. Heme is subsequently exported to the cytosol via heme transport systems, where HMOX1 cleaves the porphyrin ring (62). The released iron is either stored in the form of ferritin or exported via ferroportin into the bloodstream, where it is loaded onto its plasma carrier transferrin and delivered to erythroid precursors in the bone marrow for the synthesis of hemoglobin and other iron-containing proteins (213). However, iron is not exclusively directed to erythroid precursors, and red pulp macrophages are not the only cells involved in iron recycling. Kupffer cells, the resident macrophages of the liver, are strategically positioned within the hepatic sinusoids and play a central role in iron uptake, processing, and recycling. Sibille, Kondo, and Aisen demonstrated that these cells are capable of releasing iron in the form of ferritin, which is then rapidly taken up by hepatocytes, the primary iron-storage cells of the body. These findings highlight not only the role of liver resident macrophages in iron recycling but also a non-canonical pathway of iron transport (214). In addition, recycled iron can be supplied to other tissues, including skeletal muscle and immune cells (213). A recent study identified a subpopulation of ATMs that contains twice the intracellular iron as other ATMs and elevated expression of iron-handling genes. This population has been denoted as MFe^hi macrophages and the authors showed that they prevent iron overload of adipocytes (215). Given that iron is an essential component of numerous proteins, such as myoglobin, cytochrome P450 enzymes, components of the mitochondrial electron transport chain, and iron-sulfur cluster-containing enzymes, the role of macrophages in iron recycling is critical for the maintenance of homeostasis of virtually any tissue (**Figure 3B**).

### 4.3 Catabolism of pulmonary surfactant

Alveolar macrophages play an essential role in the metabolism of pulmonary surfactant, a lipoprotein complex that reduces surface tension at the air-liquid interface within the alveoli, thereby preventing alveolar collapse during expiration. Excess surfactant is internalized and catabolized by alveolar macrophages within lysosome-related organelles known as multilamellar bodies (216). Subsequently, free fatty acids enter fatty acid oxidation pathways or are incorporated into triacylglyceroles, while free cholesterol is either esterified for storage or exported via ABC cholesterol transporters (217). A plausible possibility is that alveolar type II epithelial cells may re-use macrophage-derived cholesterol for surfactant synthesis. However, experimental evidence supporting direct lipid transfer remains limited, as most studies have focused on the opposite direction of lipid flux (**Figure 3C**). Nonetheless, the importance of lipoprotein catabolism by alveolar macrophages is indisputable. Disruption of ABCG1 causes surfactant accumulation and pulmonary lipidosis, demonstrating that macrophage cholesterol transport is required for normal lung lipid homeostasis (218). Impaired surfactant metabolism by alveolar macrophages has also been linked to pulmonary alveolar proteinosis, chronic obstructive pulmonary disease, and idiopathic pulmonary fibrosis (219).

### 4.4 Catabolism of myelin

Similarly to degradation of excess surfactants by alveolar macrophages, astrocytes and resident macrophages of the central nervous system, microglia, actively take up and degrade myelin lipids (220). They export cholesterol onto ApoE-containing HDL-like particles in the CNS, which are transported primarily within the interstitial cerebrospinal fluid, as cholesterol in the CNS is largely separated from peripheral sources by the blood-brain barrier. These lipoproteins are subsequently recognized by receptors expressed on neurons, astrocytes, or oligodendrocytes, enabling cholesterol uptake and re−utilization for myelin formation and neuronal membrane synthesis (221). Compared with astrocytes, which are the primary source of cholesterol synthesis in the CNS, microglia contribute less to *de novo* cholesterol production. Nonetheless, experimental evidence indicates that following disruption of cholesterol biosynthesis in embryonic neurons, microglia upregulate cholesterol biosynthesis and supply it to these cells (222). In addition, microglia represent a critical source of cholesterol during remyelination (223). During this process, efficient clearance of myelin debris is essential for tissue regeneration (**Figure 3D**). Insufficient degradation of myelin lipids by macrophages can have detrimental consequences. Several leukodystrophies and neurodegenerative diseases are associated with deficiencies in macrophage lysosomal enzymes, such as galactosylceramidase in Krabbe disease, glucocerebrosidase in Gaucher disease, and sphingomyelinase in Niemann-Pick disease type A and B (224). These conditions belong to a group of more than 70 lysosomal storage diseases caused by enzyme deficiencies, transport defects, or cofactor insufficiencies. In Multiple sclerosis, macrophages and microglia engulf large amounts of myelin, which can lead to lysosomal overload, foam cell formation, impaired cholesterol efflux, and a shift toward a pro-inflammatory phenotype, ultimately contributing to defective remyelination (223, 225, 226). Microglia-mediated phagocytosis of myelin debris is therefore critical for promoting remyelination, highlighting the importance of microglial lipid recycling in restoring neural function and maintaining central nervous system homeostasis.

### 4.5 Glutamine provisioning

An important trophic role of macrophages has been observed in skeletal muscle, where regeneration is supported by infiltrating macrophages and the subsequent activation of satellite cells, the resident stem cells responsible for muscle repair and growth following injury (227). Within this tissue, macrophages produce glutamine, which is utilized by satellite cells, leading to activation of mTOR signaling pathway and the subsequent induction of proliferation and differentiation (227) (**Figure 3E**).

### 4.6 Supplementation by polyamines

Another example of macrophages providing biosynthetically costly metabolites to neighboring cells has been investigated in the intestine, where the epithelial layer undergoes continuous renewal. In this context, colonic macrophages are situated adjacent to epithelial cells and synthesize polyamines via an mTORC1–arginase-1-dependent pathway. Polyamines, such as spermine and spermidine, are subsequently utilized by these epithelial cells, promoting their proliferation. In this way, macrophages supply metabolic support to the colonic epithelium, allowing it to conserve its own metabolic precursors and thereby enhance efficient self-renewal (**Figure 3F**). The authors describe this nurturing function of macrophages as “commensal metabolism”, which becomes particularly pronounced during periods of high metabolic demand, such as inflammation-induced colitis (228, 229).

Although not yet experimentally demonstrated, a similar role of macrophages in polyamine provision may exist in adipose tissue. Brown adipose tissue contains multiple macrophage subsets, some of which are highly metabolically specialized and exhibit enhanced polyamine biosynthetic activity (230–232). During cold exposure, macrophages contribute to adaptive thermogenesis through tissue remodeling and are essential for proper thermogenic responses (233). Given their close physical interaction with adipocytes and the small, diffusible nature of polyamines, it is plausible that macrophage-mediated polyamine support in brown adipose tissue resembles the mechanism observed in the intestine. Of note, macrophages have been shown to recycle oxidatively damaged mitochondrial parts released from brown adipocytes in extracellular vesicles (230, 234).

## 5 Concluding remarks

Macrophages are classically viewed as immune sentinels specialized in pathogen clearance, yet accumulating evidence supports a more ancient and conserved role of phagocytosis in nutrient acquisition and metabolic regulation (94). We hypothesize that many trophic functions of macrophages reflect deeply conserved properties of ancestral phagocytic cells rather than independently evolved innovations. For instance, viewed through this evolutionary lens, lipid handling may represent a core component of an ancestral phagocyte toolkit. Early metazoan phagocytes exposed to lipid-rich debris would likely have required mechanisms for lipid uptake, lysosomal processing, and sterol redistribution, and elements of this metabolic machinery may have been differentially retained and elaborated across animal lineages. In taxa with strong dietary sterol dependence, such as insects, phagocytic cells including plasmatocytes may therefore play particularly important roles in sterol recovery and distribution. In our view, these observations are more parsimoniously explained as lineage-specific elaborations of an ancestral phagocyte-associated metabolic program than as independent examples of convergent evolution. Consistent with this interpretation, mammalian macrophages retain robust lipid-processing capacities that underlie their roles in cholesterol handling, recycling, and systemic lipid homeostasis. Recognizing that these ancient functions are deeply embedded within phagocytic biology raises the possibility that they remain underappreciated in current immunological frameworks and suggests that future studies adopting this perspective may uncover additional examples of macrophage involvement in organismal metabolism.

Comparative insights from invertebrates further suggest that nutrient recycling and trophic support represent ancestral functions of phagocytic cells that persist in tissue-resident macrophages in mammals (94, 235, 236). Tissue-resident macrophages are strategically positioned to regulate local nutrient availability through the uptake, storage, processing, and release of metabolites, lipids, and biosynthetic precursors. In this context, tissue-resident macrophage subpopulations may provide essential but often overlooked trophic and metabolic support to surrounding cells and tissues. Failure of these functions could impair tissue repair and regeneration, compromise stem cell niches, or alter the metabolic fitness of neighboring cells. Conversely, enhancing macrophage-mediated nutrient recycling may represent an underexplored therapeutic strategy for restoring tissue homeostasis in degenerative and metabolic diseases.

Future work should therefore define the molecular pathways linking cargo composition to macrophage fate and function using integrated experimental approaches. Metabolic tracing studies employing isotope-labeled lipids, amino acids, or apoptotic cargo will be essential for determining which metabolites derived from engulfed material are retained, catabolized, or recycled into new biomolecules. Combining these approaches with single-cell transcriptomics, epigenomics, spatial metabolomics, and proteomics could reveal how distinct cargo types reprogram macrophage states across tissues and disease contexts. *In vivo* lineage-specific perturbation models targeting lysosomal transporters, lipid-handling pathways, or metabolite export machinery will be particularly important for determining whether macrophage-derived nutrients directly support neighboring cells. Another major challenge will be to determine how cargo-derived metabolites influence chromatin state, transcription factor activity, and long-term macrophage identity. We predict that disrupting macrophage-mediated nutrient recycling will impair tissue homeostasis and promote metabolic disease, whereas enhancing these pathways may provide therapeutic benefit.

Importantly, these concepts may have broad medical relevance. Many chronic non-communicable diseases are characterized by defective handling of cellular debris and altered lipid or nutrient metabolism, processes in which macrophages are centrally involved. In atherosclerosis, macrophages internalize large quantities of lipid-rich cargo and can either promote tissue homeostasis through cholesterol efflux and metabolic recycling or contribute to pathology through foam cell formation, inflammasome activation, and necrotic core development. From the perspective proposed here, these pathological states may reflect maladaptive or overwhelmed ancestral nutrient-processing programs. Similarly, in obesity and metabolic syndrome, adipose tissue macrophages are exposed to excessive lipid flux and dying adipocytes, potentially altering nutrient-recycling pathways and contributing to chronic low-grade inflammation, insulin resistance, and tissue remodeling. In neurodegenerative disorders, including Alzheimer’s disease and related proteinopathies, microglia may likewise fail to efficiently process or redistribute metabolites derived from engulfed neuronal debris, thereby contributing to defective proteostasis, altered lipid metabolism, and progressive neuroinflammation. This framework further suggests that macrophage dysfunction in disease may not arise solely from aberrant inflammatory signaling but also from disruptions in the trophic and metabolic functions normally coupled to phagocytosis.

Overall, integrating immunological, evolutionary, and metabolic perspectives may reveal that macrophages function not only as immune effectors but also as central coordinators of tissue nutrient economy.
